# Food Trying and Liking Related to Grade Level and Meal Participation

**DOI:** 10.3390/ijerph17165641

**Published:** 2020-08-05

**Authors:** Jennifer Hanson, Janelle Elmore, Marianne Swaney-Stueve

**Affiliations:** 1 Department of Food, Nutrition, Dietetics and Health, Kansas State University, Manhattan, KS 66506, USA; 2 Elmore Consulting, Columbia, MO 65202, USA; je@sensoryhelp.com; 3 Sensory & Consumer Research Center, Kansas State University, Manhattan, KS 66506, USA; marianness@ksu.edu

**Keywords:** emoji, food preferences, food selection, fruit, students, surveys and questionnaires, vegetables

## Abstract

School-based child nutrition programs provide students with meals and snacks that align with guidelines for a healthy eating pattern. However, participation is not universal, and research on the determinants of food selection is needed to improve school nutrition practices and policies. The purpose of this study was to examine the relationships between grade level (i.e., grade school, middle school, or high school) as well as meal participation category (i.e., only breakfast, only lunch, or both) and food trying and liking in a large urban school district. Outcomes were measured using an online survey completed by students from 2nd through 12th grade (*n* = 21,540). Breakfast and lunch item liking scores were higher among the grade school and middle school students than among the high school students. Breakfast and lunch liking scores were also higher among those who participated in both breakfast and lunch as opposed to those who only participated in one meal. Food item liking scores were positively correlated with the percentage of students who had tried the particular foods (*r* = 0.52, *p* < 0.001), and the number of foods tried was dependent on both grade level and meal participation category (F(4, 21,531) = 10.994, *p* < 0.001). In this survey of students, both grade level and meal participation category were found to be related to the liking of foods, while foods that were tried more often tended to be liked more. Future studies should consider grade level and meal participation when exploring student preferences. School nutrition programs should also consider these factors when assessing satisfaction.

## 1. Introduction

Optimal growth and development is reliant on sound nutrition [1,2,3]. Yet, the diets of many children and adolescents in the United States (U.S.) do not meet nutrient recommendations [4] and fall short of current guidelines for a healthy eating pattern [5]. Behaviors that contribute to dietary shortfall among children (e.g., consumption of sugar-sweetened beverages, limited fruit and vegetable intake) are associated with an array of individual and environmental factors including infant/child feeding practices [6,7,8,9], food neophobia [6,8], parental eating patterns [6,9,10], food insecurity [11], television watching [9,12], the food environment [9,10], and public policies [13]. 

Schools provide numerous opportunities to influence long-term food habits, thereby making them a prime environment for improving the eating patterns of students [14]. The U.S. Department of Agriculture (USDA) school-based child nutrition programs are important public programs developed to ensure that children receive meals and snacks that align with current guidelines for a healthy eating pattern [15]. The National School Lunch Program (NSLP), which serves nearly 30 billion meals per day, is the second largest food and nutrition assistance program in the U.S. [15]. Other school-based child nutrition programs include the School Breakfast Program (SBP), the Fresh Fruit and Vegetable Program, and the Special Milk Program [15]. 

While these USDA child nutrition programs provide many benefits, participation is not universal [16]. Furthermore, among students participating in school meal programs, food acceptance and food waste is an ongoing issue [17,18,19,20,21,22]. In addition, satisfaction, in particular taste, is a common reason that students cite for throwing away food [17]. Needless to say, research on the determinants of food selection and food satisfaction has important implications for school nutrition practices and policies. The role of initiatives such as choice architecture [23], motivational incentives [22,23], and the Farm to School Program [19,24] has been explored in a variety of manners. The relationships between socioeconomic and demographic characteristics and school nutrition program outcomes have been studied as well [25,26,27,28]. However, in all but a few exceptions, the underlying effects of grade level (i.e., grade school, middle school, or high school) and meal participation category (i.e., only breakfast, only lunch or both) have been overlooked.

There are important socioeconomic differences associated with school nutrition program participation. NSLP participation rates vary among students based on free, reduced-price, and paid eligibility category [29]. In addition, the NSLP and the SBP differ with regard to both the number of meals served and the proportion of participants who qualify for free or reduced-price meals. The SBP serves fewer meals, but of the meals served, a higher proportion is provided to students who qualify for free and reduced-price meals [30]. Compared to students who pay full price, students receiving free or reduced-price meals are less likely to select a vegetable and more likely to select milk [25]. In addition, students paying full price are more likely to bring lunch from home and more likely to select school entrées higher in protein [27]. In turn, these differences may result in food preferences that are associated with meal participation category. 

In studies designed to examine food consumption and plate waste, grade-level differences in whole fruit [18], vegetable [21], grain [21], and protein food [21] consumption have been noted. A study of school children’s food preferences by Cooke and Wardle [31] found age-related differences in the number of foods tried and the number of foods liked. A survey conducted within an Ohio school district revealed food preference differences among elementary, middle and high school students [32]. However, this survey of students in Ohio was conducted prior to the implementation of the Healthy, Hunger-Free Kids Act of 2010 (HHFKA) [33], and many of the most-liked foods were high in fat and calories. Since implementation of the HHFKA, school meal programs have had to adopt a number of changes, which have resulted in the reduction of saturated fat [34] and sodium [34,35] and improved overall diet quality [35]. However, these changes have also resulted in modifications to, or the disappearance of, various familiar foods [36]. The purpose of this study was to examine the relationship between grade level as well as meal participation category (i.e., only breakfast, only lunch or both) and food trying and liking in a large urban school district following the implementation of the HHFKA.

## 2. Materials and Methods 

### 2.1. Participants

Responses were analyzed from a volunteer sample (*n* = 21,540) of school children from one urban school district in the U.S. The children, students from 2nd through 12th grade, all participated in the meal service (i.e., breakfast, lunch, or breakfast and lunch). 

### 2.2. Survey

The survey was developed in cooperation with the school district’s nutrition department and the food service management firm responsible for providing meals to the district’s schools. The survey was designed to collect data on a variety of aspects including dining experience satisfaction, food selection and liking, least and most liked condiments, favorite foods (e.g., Mexican food, Asian food, soul food/traditional southern food) and flavoring profiles, willingness to try different items, and preference for various diets or eating patterns (i.e., vegan, Kosher, Halal). This study was limited to a set of items regarding food selection and liking. The food liking items included 48 core school foods which were comprised of 23 breakfast items and 25 lunch items. Before being asked about food item liking, students were first asked to indicate which of the core school foods they had previously eaten. Liking responses were only collected for foods that were eaten. In addition to the core items, all students rated their liking of fruit as a broad category, cold vegetables as a broad category, and hot vegetables as a broad category. Individual breakfast and lunch core items included a pictorial prompt of the food items, while the fruit, cold vegetable and hot vegetable categories included a graphic illustrating a variety of items. As a method to establish a benchmark for liking beyond school food items, students who indicated that they brought food from home were asked to rate their liking of food from home. 

Students indicated their liking rating for each of the individual food items and broad food categories using a 7-point emoji facial scale [37]. This emoji scale was developed as an alternative to the Peryam and Kroll (super good/super bad) 9-point hedonic scale for children [38]. Findings from a comparison study [37] suggest the emoji scale is a suitable (and more modern) option. Prior to being utilized in the current study, the 7-point emoji facial scale was incorporated into preliminary versions of the survey that were pilot tested once in 2017 and again in 2018. In both cases, the proportion of surveys left incomplete was not deemed excessive. 

For analysis purposes, the response options were coded 1 through 7, with the most negative emoji being coded as a 1 and the most positive emoji being coded as a 7. Liking for a food was defined as a score of 6 or 7, and disliking as a score of 1 or 2. For the Cronbach’s alpha analysis, “never tried” was scored a 0.

It should be noted that the demographic information solicited from student participants was limited to grade level. While gender, ethnicity or other socio-demographic information are commonly solicited in surveys, the school district considered these questions sensitive in nature, so they were specifically excluded from the survey. 

### 2.3. Implementation

All schools in the district were eligible to participate. The school food service team worked with school administrators to distribute an anonymous survey link to their student population (*n* = 361,314). The survey was completed either as a classroom activity or during the students’ personal time. Based on a review of time stamps, a majority of the surveys were completed during the school day and seemed to cluster time-wise within a building. Of the 24,767 surveys that were completed, 21,540 surveys from 449 school buildings contained the completed responses necessary for inclusion in this study. Information regarding whether the survey was read aloud for those children who had difficulty reading was not obtained. The survey was noninvasive and voluntary. Students and their parents were not required to complete consent forms. The Kansas State University Institutional Review Board approved the research protocol (IRB proposal #5930). The survey was a self-administered, online survey using Compusense Cloud (Compusense, Inc., Guelph, ON, Canada) available from 13 January to 22 March 2019.

### 2.4. Statistical Analysis

Statistical analysis was performed using the IBM SPSS Statistics for Windows, Version 25 (IBM SPSS Statistics for Windows, IBM Corporation, Armonk, NY, USA). For all analyses, the significance level was identified at *p* < 0.05. Correlation analysis examined the relationship between the percentage of children indicating they had eaten each core food and the mean liking score of those who had eaten it. Participation in school meals was categorized as one of three levels: breakfast only, lunch only, or breakfast and lunch. Three grade level groupings were created: elementary school, 2nd through 5th grades; middle school, 6th through 8th grades; and high school, 9th through 12th grades. Univariate ANOVA was used to examine the effects of grade level, meal participation category and grade level-by-meal participation category interaction on the number of foods tried, while ANCOVA was conducted to determine the impact of the same main effects and interaction on the number of foods liked or disliked, controlling for the number of foods tried. 

The core food items were divided into two groupings, breakfast (23 items) and lunch (25 items), to be assessed by Cronbach’s alpha. Further analysis investigated the effect of grade level and meal participation level on mean liking scores for each category of foods.

## 3. Results

Survey responses were obtained from elementary (*n* = 9731), middle (*n* = 8668), and high school (*n* = 3141) students. Respondents had tried, on average, fourteen of the 48 core school food items. 

### 3.1. Liking Ratings

Across the sample, the five most highly rated breakfast items were tator tots (*M* = 6.12), strawberry and yogurt parfait (*M* = 5.90), French toast sticks (*M* = 5.76), waffles (*M* = 5.72) and cereal (*M* = 5.69). Of these five items, tator tots, strawberry and yogurt parfait, and French toast sticks were among the top five most highly rated items for each grade level. Overall, the five most highly rated menu items for lunch were yogurt and cheese kit (*M* = 5.83), spicy popcorn chicken (*M* = 5.64), nachos (*M* = 5.62), chicken nuggets (*M* = 5.46) and cheese pizza sticks with marinara sauce (*M* = 5.38). All but chicken nuggets and cheese pizza sticks with marinara sauce were among the most highly rated for each grade level. 

Among all students, the five breakfast items with the lowest ratings were egg and cheese on English muffin (*M* = 4.68), pear and yogurt parfait (*M* = 4.67), strawberry kiwi bar (*M* = 4.60) oatmeal raisin bars (*M* = 4.58), and egg and cheese quesadilla (*M* = 4.28). All but egg and cheese on English muffin and oatmeal raisin bars were consistently among the bottom five menu items for each grade level. Overall, the lunch items with the lowest ratings were cheesy nacho bake (*M* = 4.53), cheese ravioli (*M* = 4.42), peanut and butter jelly sandwiches (*M* = 4.42), burritos (*M* = 4.38) and tuna melt (*M* = 3.91). Of these lunch items, cheesy nacho bake and cheese ravioli were among the lunch items with the lowest rated items for each grade group.

### 3.2. Number of Foods Tried

**Figure 1:**
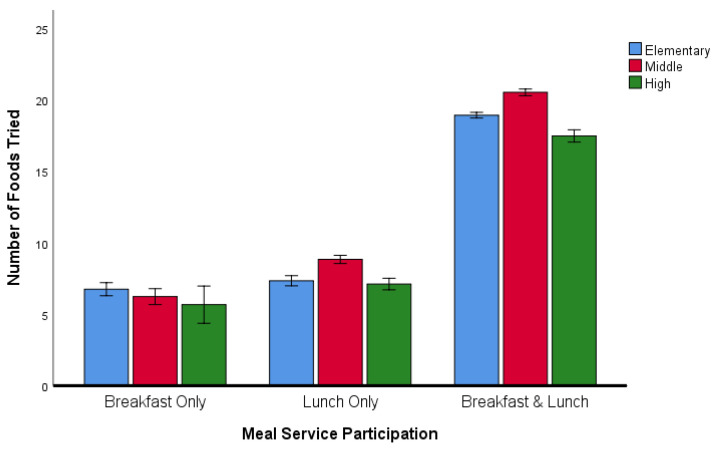
Number of foods tried by different grade groups within meal service participation level. Values are means with 95% CI indicated by vertical bars.

Across the sample, liking scores for each of the core items were positively correlated with the percentage of students who had tried the particular foods (*r* = 0.52, *p* < 0.001). ANOVA revealed a statistically significant interaction between the effects of grade level and meal participation category on the number of foods tried (F(4, 21,531) = 10.994, *p* < 0.001). The mean number of items tried with 95% confidence intervals (CI) are presented in Figure 1. The number of foods tried was greatest among students participating in both breakfast and lunch meal service. The number of foods tried did not vary with grade level among students who only participated in the breakfast meal service. However, among students who participated in lunch only, as well as among those who participated in both breakfast and lunch, middle school students tried more food items than either the elementary or the high school students.

### 3.3. Number of Foods Liked

**Figure 2:**
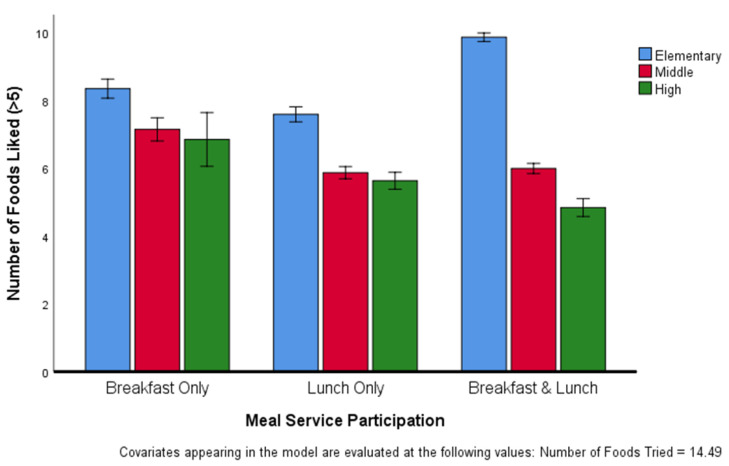
Number of foods liked, adjusted for number of foods tried, by different age groups within meal service participation. Values are means with 95% CI indicated by vertical bars.

The ANCOVA controlling for the number of foods tried revealed a significant two-way interaction between grade level and meal participation (F(4, 21,530) = 84.651, *p* < 0.001). The mean number of foods liked with 95% CI is presented in Figure 2. Within each meal participation category, elementary students liked more food items than any other grade level. Among students who only ate breakfast as well as among those who only ate lunch, middle school and high school students liked a similar number of items, while high school students participating in both breakfast and lunch liked fewer items than the middle school students in the same participation category. 

### 3.4. Number of Foods Disiked

**Figure 3:**
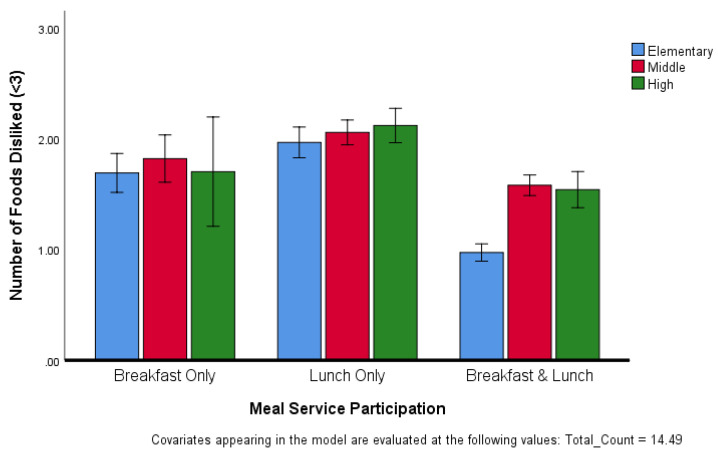
Number of foods disliked, adjusted for number of foods tried, by different age groups within meal service participation. Values are means with 95% CI indicated by vertical bars.

As with the number of food items liked, ANCOVA revealed that the number of food items disliked was dependent on both grade level and participation category (F(4, 21,530) = 7.759, *p* < 0.001) (Figure 3). The number of items disliked did not vary significantly among grade level groups who participated in breakfast only or lunch only. However, among students who participated in both breakfast and lunch meal service, high school and middle school students disliked more items than elementary students. 

### 3.5. Satisfaction Scale

To examine the pattern of food preferences, core menu item scores were grouped by meal. Cronbach’s alphas were 0.85 and 0.86 for breakfast and lunch, respectively. Category-based satisfaction scales were produced for both breakfast items and lunch items by calculating the mean of the liking scores of the foods in each category. Item means for both the breakfast and lunch scales along with the mean liking for food from home, fruit, hot vegetables and cold vegetables (by grade group and participation level) are presented in Table 1. 

Across all school groups, food from home was most well-liked, followed by breakfast items, fruit, lunch items and lastly vegetables (cold and hot). Age-related differences in liking were observed for both meal item scales (i.e., breakfast and lunch) and individual items (i.e., food from home, fruit, cold vegetables, and hot vegetables). Liking was highest among the youngest students for both breakfast and lunch scales and all individual food items, except food brought from home, where liking was highest among middle school students. Liking was lowest among the oldest students for both scales and all individual items with two exceptions, cold and hot vegetables. For these two items, liking was lowest among middle school students.

Lunch, fruit and vegetables (hot and cold) were most well-liked by students who participated in both the breakfast and lunch meal service, while liking was highest for food from home and breakfast among students who only participated in the breakfast meal service. Liking was lowest for food from home, lunch items and fruit among students that participated in lunch meal service exclusively. Liking for vegetables (cold and hot) was lowest among the students who only participated in the breakfast meal service. Finally, liking for breakfast items was lowest among students who ate both breakfast and lunch provided meals.

## 4. Discussion

In this survey of students from 2nd through 12th grade, grade level and meal participation category, were found to be related to both the trying and the liking of foods. In addition, we observed that foods that were tried more often tended to be liked more. In all, these findings add to the overall understanding of meal service outcomes and in turn have important implications for school nutrition practices and policies.

Aside from the group of students who only ate breakfast, the number of food items tried increased with grade level for those in elementary through middle school. This finding is similar to that obtained by Cooke and Wardle [31], who found that the number of foods tried increased with age in their study of school children. However, contrary to what might be expected, the number of foods tried decreased with grade level for those in middle through high school. This observation may be due in part to the timing of the menu changes that accompanied the implementation of the HHFKA. The high school students in this study were in elementary school at the onset of the implementation of the HHFKA. Those that were in middle school were either not yet in school or were in the very early elementary grades (e.g., kindergarten, 1st grade) when implementation of the HHFKA began. As such, the high school students had greater exposure to the pre-HHFKA school foods, and therefore may have been reluctant to try the new healthier menu items. The middle school students, some of whom started school after implementation of the HHFKA, had less exposure to the pre-HHFKA school foods. Having known little else, these middle school students may have been more willing to try the menu items. 

The association between grade level and food liking uncovered here is in line with previous research in which ratings of many food types differed by age or grade level [31,32]. However, whereas school food liking was the highest among the elementary school students in the current study, the liking of most entrée food groupings was highest among high school students in an earlier study [32].

The most well-liked item was food from home, and students have previously indicated a preference for food from home as a reason for not eating school lunch [39]. These observations are concerning, as Farris et al. [40] noted that the majority of lunches from home include a dessert item, many include sugar-sweetened beverages, and a large number are missing both fruits and vegetables. Highly rated lunch items in this study were similar to those found to be well liked in an earlier study (i.e., pizza, chicken nuggets, yogurt, and cheese) [32]. Several of the lunch items with the lowest ratings were also similar to those with lower ratings in the same earlier study (e.g., peanut butter and jelly sandwich, tuna salad sandwich, burrito) [32]. Students have cited appearance, quality, and taste [39] as factors impacting their eating decisions, and efforts should be made to determine what factors are behind the low ratings of the less liked items in this study. 

Vegetables were the least liked menu items, with hot vegetables receiving lower ratings than cold vegetables. Aside from cooked potato items, vegetables were not found to be a favorite among school children in an earlier study [32]. Vegetables as a whole have received low ratings [31] and tend to be the most wasted category of school foods [17,20]. The liking of vegetables in this current study was lowest among middle school students. Conversely, fruit liking ratings were highest among the elementary school students, and this finding is supported by an earlier observation in which fruit was more positively rated by elementary students as opposed to the older middle school and high school students [32]. These findings regarding fruit are not surprising, as children 4- to 8 years of age consume a larger portion of their total intake as fruit compared to those 9- to 13 years of age as well as those 14- to 18 years of age [5]. 

On average, each student had tried less than one-third of the core food items listed on the survey. This relatively low rate of trying is concerning and may be a result of the overall participation rate. As was reported previously by Cook and Wardle [31], a positive correlation was observed between food item liking and the proportion of students who tried a food in this current study as well. Providing students with an opportunity to try menu items by way of taste testing events or product sampling can increase exposure and may, in turn, increase the acceptance of menu items. Data from an earlier study of school children revealed that some students participate in school meals regularly, while others participate rarely [27]. Offering opportunities to taste menu items may be a way in which to increase exposure, especially among students who do not participate regularly. Given that trying and liking are associated, opportunities to try school menu items may also be an approach to increase overall participation. Likewise, the local procurement of produce, which has been shown to positively affect vegetable consumption, may increase the trying and liking of foods [24]. Lastly, non-food motivational incentives such as stickers and stamps may also encourage positive eating behaviors [22,23]. However, the effect of such awards appears to vary by country [22], and as Decosta et al. [23] pointed out, incentives should be used with caution, as additional “research is needed to examine how instrumental feeding might affect children’s intrinsic drive to explore novel food”. 

Strengths of this study include the large sample size and the post-HHFKA timing of the survey, which was seven years after the initial implementation of the HHFKA. Willingness to try a given food [41] and food liking [42,43] are known to increase with repeated exposure. Preference ratings such as ours, which were collected after the initial HHFKA-adjustment period, provide insight beyond that which was obtained during the early phases of implementation. The uncovering of a relationship between meal participation and food preferences is novel and adds to the body of knowledge. 

While significant, many of the differences in liking scores may be considered small. However, one will note that the range for mean liking scores was relatively narrow at 1.84 for breakfast item scores and 1.92 for lunch item scores. In addition, of the 25 lunch items, the lowest scoring “top five” item was cheese pizza sticks with marinara sauce (*M* = 5.38), while the highest scoring “bottom five” item was cheesy nacho bake (*M* = 4.53). The difference between the mean values for these lunch items was 0.85. Similarly, for the 23 core breakfast items, the lowest scoring “top five” item was cereal (*M* = 5.69), while the highest scoring “bottom five” item was egg and cheese on English muffin (*M* = 4.68) with the difference between these values at 1.01. 

Understandably, larger differences in liking have greater “real-world” meaning for school nutrition programs. Nonetheless, establishing a meaningful size cut-point for these differences is difficult, and this is particularly so when ordinal scales (e.g., Likert scales) are utilized. As such, the number of students impacted and the decisions at stake should all be taken into consideration when establishing cut-points. Furthermore, because this was an observational study, causal relationships cannot be inferred. For school nutrition programs, interpretations of our results should be made with this limitation and the broader body of knowledge in mind. 

Although beyond our control, the inability to collect demographic information beyond grade level and meal participation category is a limitation of this study. Reliance on a volunteer sample from one school district is an additional limitation which constrains generalizability to other schools. In addition, students completed the survey in a variety of environments (i.e., at home, at school during class or at school on their own time), and measures were not in place to prevent students from taking the survey more than once. Although, given that the average time to complete the survey was approximately 12 min, the occurrence of multiple survey submissions was unlikely. 

## 5. Conclusions

School nutrition programs offer many benefits, but program success is dependent on a variety of factors. The timing and the length of the lunch period, school policies, and parental perceptions, are all associated with school nutrition program outcomes. In this survey of students, both grade level and meal participation category were found to be related to the trying and the liking of foods. While additional research is needed, the results of this study build upon that which is already known about the relationship between socioeconomic and demographic characteristics and child nutrition program outcomes. Future studies should consider the inclusion of both grade level and meal participation category when exploring student preferences. School nutrition programs should also consider these factors when assessing student satisfaction. 

## Figures and Tables

**Table 1:** Mean liking ^1^ of items in each food category ^2^ (by grade group and meal service participation).

	**Grade Group**									
	**Elementary**			**Middle**			**High**			
**Item**	**Mean**	***n***	**SD**	**Mean**	***n***	**SD**	**Mean**	***n***	**SD**	***p*-Value**
Food from home	6.35 a	6450	1.08	6.38 a	5076	0.98	6.23 b	1610	1.00	*p* < 0.001
Breakfast	5.80 a	7659	1.09	5.14 b	5537	1.21	5.05 c	1531	1.19	*p* < 0.001
Lunch	5.60 a	8485	1.24	4.87 b	7834	1.29	4.61 c	2983	1.25	*p* < 0.001
Fruit	5.71 a	9731	1.55	4.93 b	8668	1.62	4.91 b	3141	1.53	*p* < 0.001
Cold vegetables	3.82 a	9731	2.09	3.48 c	8668	1.81	3.71 b	3141	1.76	*p* < 0.001
Hot vegetables	3.23 a	9731	2.01	2.74 c	8668	1.68	2.98 b	3141	1.69	*p* < 0.001
	**Participation Level**									
	**Breakfast only**			**Lunch only**			**Breakfast and Lunch**			
**Item**	**Mean**	***n***	**SD**	**Mean**	***n***	**SD**	**Mean**	***n***	**SD**	***p*-Value**
Food from home	6.44 a	1651	1.03	6.26 c	3725	1.05	6.37 b	7760	1.02	*p* < 0.001
Breakfast	5.58 a	2209	1.19	na	na	na	5.45 b	12518	1.20	*p* < 0.001
Lunch	na	na	na	4.97 b	6737	1.34	5.24 a	12565	1.30	*p* < 0.001
Fruit	5.08 b	2231	1.77	5.05 b	6470	1.57	5.44 a	12569	1.61	*p* < 0.001
Cold vegetables	3.41 c	2231	1.99	3.57 b	6470	1.82	3.77 a	12569	1.99	*p* < 0.001
Hot vegetables	2.76 c	2231	1.82	2.85 b	7113	1.71	3.11 a	12569	1.92	*p* < 0.001

^1^. Response scale for each item is 1–7, 1 (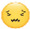), 2 (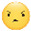), 3 (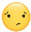), 4 (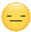), 5 (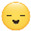), 6 (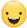), 7 (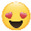). ^2^. Mean values within a row with unlike letters were significantly different (*p* < 0.05).
